# Loss of consciousness associated with Shime-Waza in judo: an exploratory study of coaches' perceptions, experiences, and practices

**DOI:** 10.3389/fspor.2026.1955805

**Published:** 2026-10-23

**Authors:** Emily Allen, Kabir Singh, Wieslaw Blach, Akira Ikumi, Eiji Sasaki, Sercan Illy, Manuela Angioi, Nikos Malliaropoulos, Filippo Migliorini, Nicola Maffulli

**Affiliations:** 1 Centre for Sports and Exercise Medicine, William Harvey Research Institute, Queen Mary University of London, London, United Kingdom; 2 University of Leeds, Leeds, United Kingdom; 3 Faculty of Sport, University School of Physical Education in Wroclaw, Wrocław, Poland; 4 Department of Orthopaedic Surgery, University of Tsukuba, Ibaraki, Japan; 5 Department of Orthopaedic Surgery, Hirosaki University Graduate School of Medicine, Aomori, Japan; 6 Department of Physiotherapy, University of Hacettepe, Ankara, Türkiye; 7 Sports Clinic, Rheumatology Department, Barts Health NHS Trust, London, United Kingdom; 8 Sports and Exercise Medicine Clinic, Thessaloniki, Greece; 9 Department of Trauma and Reconstructive Surgery, University Hospital Halle, Martin Luther University Halle-Wittenberg, Halle (Saale), Germany; 10 Department of Life Sciences, Health, and Health Professions, Link Campus University, Rome, Italy; 11 Department of Orthopaedic and Trauma Surgery, Eifelklinik St. Brigida, Simmerath, Germany; 12 Department of Trauma and Orthopaedic Surgery, Faculty of Medicine and Psychology, University La Sapienza, Roma, Italy; 13 School of Pharmacy and Bioengineering, Keele University, Faculty of Medicine, Stoke-on-Trent, United Kingdom

**Keywords:** choking techniques, combat sports, judo, loss of consciousness, martial arts

## Abstract

**Objectives:**

Loss of consciousness (LOC) can occur in judo following shime-waza (strangling techniques) in the age and competition categories in which these are permitted. Acute physiological mechanisms of LOC in this context have been investigated, but the consequences of repeated exposure remain uncertain. This exploratory study examined coaches' perceptions, awareness, and self-reported practices concerning LOC associated with shime-waza.

**Methods:**

A study-specific questionnaire was distributed through judo clubs in the United Kingdom, Greece, and Japan. Eligible adult coaches were invited to report their experiences in dealing with LOC induced by shime-waza, including around its prevention and management. The questionnaire was developed by an expert panel and pilot-tested for usability. We report descriptive frequencies and percentages using item-specific denominators.

**Results:**

Twenty-three male coaches (mean age 47.2 years, range 21-76 years; median coaching experience >20 years) participated, of whom 17/23 were United Kingdom-based. Most reported observing LOC less than monthly or never (21/23) and recalled it more often in competition than in training (15/23). Perceptions of potential consequences, estimated duration, and similarity to concussion were heterogeneous. Eighteen coaches (78.3%) reported awareness of an LOC protocol, whilst perceptions of medical support and post-event behavioural change also varied.

**Conclusion:**

Coaches reported varied experiences and practices regarding LOC associated with shime-waza. The findings are exploratory and cannot quantify knowledge, LOC incidence, or the effectiveness of current safety practices. However, they identify areas where further coach education may be useful, support appropriate safeguards, prompt recognition and assessment of LOC, and support larger studies using validated instruments and exposure-based data.

## Introduction

Judo is a globally practised combat sport comprising standing techniques (tachi-waza) and groundwork (ne-waza) (1). Within ne-waza, athletes may establish control through osaekomi-waza (holding techniques) or seek submission through shime-waza (strangling techniques) and kansetsu-waza (joint-locking techniques) (2). The teaching and use of shime-waza are progressive and restricted by practitioners' age and experience level.

The Kodokan Judo Institute (Kodokan), founded by Jigoro Kano and regarded as the founding institution of judo, provides the traditional technical nomenclature for shime-waza and lists 12 recognised techniques (3). Examples include hadaka-jime (rear-naked choke) and gyaku-juji-jime (reverse cross strangle) (4). These techniques use the arms, legs, or uniform collar to apply compression to the neck of an opponent (5). Players may signal submission verbally or non-verbally ('tap out’), at which point the technique should be released immediately. A referee may also stop a contest where a player is unable to continue safely, including after loss of consciousness (LOC).

Acute LOC following shime-waza is most plausibly related to transient cerebral hypoperfusion resulting from vascular neck compression. Carotid compression, impaired venous outflow, airway compromise, and reflex cardiovascular responses may also contribute (4, 6–8). Early physiological research and subsequent experimental studies have examined these acute effects; however, the relative contribution of each mechanism in a single event remains uncertain (9, 10). LOC has been reported to occur within 10 to 20 s of effective strangulation, with recovery of consciousness usually occurring shortly after release (6, 11). These timings should be interpreted cautiously as they may vary by technique and circumstance.

Whilst serious adverse events in supervised sport appear uncommon, rapid apparent recovery after LOC does not imply the complete absence of harm from repeated exposure (12). Critically, the current evidence base remains insufficient to determine long-term neurological risk. This differs from LOC associated with traumatic brain injury (TBI), which may follow a throw, a fall, or collision, and has a distinct injury mechanism and established potential for longer-term neurological sequelae (13). In this context, player safety encompasses appropriate technical instruction, appropriate age-adjusted practice, prompt recognition of LOC, access to medical assessment, incident reporting, and careful return to activity. Safety in judo also relies on education, safeguarding, responsible coaching, and evaluation of practice (14).

Despite a growing literature on judo, evidence specifically concerning shime-waza remains limited. This exploratory questionnaire study described coaches' perceptions, self-reported experiences, awareness, and practices surrounding LOC associated with shime-waza, including its perceived consequences, prevention, and management.

## Methods

Ethics approval was granted by the Queen Mary Ethics of Research Committee, Queen Mary University of London (QMREC2018/48/092). All participants provided written informed consent before data collection. The study was conducted in accordance with the Declaration of Helsinki.

### Questionnaire design

We conducted an exploratory questionnaire study using a sample of judo coaches. The study was designed to describe participant-reported perceptions, experiences, awareness, and practices. It was not designed to measure an objective level of knowledge or to estimate the incidence or prevalence of LOC.

A five-member panel with judo and sports medicine expertise developed the questionnaire: (i) an academic, judo coach, and 7th dan in judo; (ii) a consultant sports physician, judo coach, and 6th dan; (iii) an orthopaedic surgeon, national judo medical director, and 5th dan; (iv) an orthopaedic surgeon and national judo team physician; and (v) an elite judo athlete and 2nd dan. No formal psychometric validation was undertaken. Members independently reviewed draft items and met to agree the final content of the questionnaire. The questionnaire used the non-standard descriptive labels ‘collar-used’, ‘arm-used’, and ‘leg-used’ to describe the principal means of applying a technique; these labels are not a formal Kodokan classification, and do not imply differential objective risk.

The English-language questionnaire comprised two thematic sections: (i) perceptions and experiences of LOC and its consequences; and (ii) awareness, perceptions, and practices relating to prevention and management. Participant age, gender, location, coaching qualification, experience, coaching level, and judo rank were collected. Some items permitted optional free-text comments. These comments were retained as contextual material but were not systematically analysed and did not contribute to reported quantitative findings or interpretative claims. They are therefore not presented separately in the Results.

A pilot survey of six judo coaches assessed usability and wording. Neither the expert review nor pilot testing constituted formal content-validity, reliability, or construct-validity testing. Therefore, the questionnaire should be regarded as study-specific and non-validated. A blank questionnaire is supplied as Supplementary Material.

### Participant recruitment and data collection

Judo clubs in the United Kingdom, Greece, and Japan were contacted by email and asked to circulate the study invitation amongst their coaches. Recruitment was convenience-based and the number of coaches who received an invitation was not recorded. The sample must therefore not be interpreted as internationally representative. Eligible participants were active (currently delivering judo coaching at the time of recruitment) adult judo coaches who self-reported at least a UK Coaching Certificate (UKCC) Level 2 qualification, or a qualification judged broadly equivalent within their national system. UKCC Level 2 was used as a pragmatic minimum formal coaching-qualification threshold. As coaching pathways vary internationally and have evolved over time, equivalence of non-UK qualifications was based on self-report and could not be independently verified. Because the questionnaire was available only in English, participants also required sufficient written and verbal English proficiency to complete it.

Interested coaches contacted the research team by return email and were sent the participant information, consent materials, and questionnaire. Participants returned completed questionnaires after written informed consent was obtained. No formal *a priori* sample-size calculation was performed. The original expectation that approximately ten clubs would contribute around three eligible coaches each was a feasibility-based recruitment target, not a precision- or power-based sample-size justification.

### Statistical analysis

We performed descriptive quantitative analyses. For each closed-response item, we report the number of valid responses and percentage, using the number of non-missing responses to that item as the denominator. Free-text responses were retained as contextual material but were not systematically analysed or used to generate reported findings or interpretative claims. Formal qualitative coding framework, inter-rater reliability assessment, or inferential statistical testing was not undertaken.

## Results

Twenty-three coaches participated in the study. Seventeen (73.9%) were from the United Kingdom, two (8.7%) were from Greece, one (4.3%) was from Japan, and three (13.0%) did not specify their location on the questionnaire. All participants were male. The mean age was 47.2 years (range 21-76 years), and the median reported coaching experience was >20 years. Qualifications, coaching levels, and rank are presented in Table 1. Exact qualification information was incomplete or heterogeneous for some participants.

**Table 1:** Participant characteristics.

	Age (years)	Location	Coaching qualification	Experience (years)	Coaching level
1	29	United Kingdom	UKCC/BJA level 2	6-10	National
2	62	United Kingdom	UKCC/BJA level 3	>20	Club, regional, national, international
3	61	United Kingdom	UKCC/BJA level 2	>20	Club, regional, national
4	55	United Kingdom	UKCC/BJA level 2	11–20	Club, regional, national, international
5	27	United Kingdom	UKCC/BJA level 2	6–10	Club, regional, national
6	35	United Kingdom	UKCC/BJA level 2	6–10	International
7	76	United Kingdom	UKCC/BJA level 3	>20	Club, regional, national, international
8	60	United Kingdom	UKCC/BJA level 2	>20	National
9	56	United Kingdom	EJU level 5	>20	International
10	48	United Kingdom	UKCC/BJA level 4, EJU level 3, IJF academy	>20	Club, regional, national, international
11	57	United Kingdom	UKCC/BJA level 2	6–10	Club, regional, national
12	45	United Kingdom	EJU Level 4	>20	International
13	21	Greece	Not specified	<1	International
14	25	Greece	Aristotle University of Thessaloniki	<1	National
15	61	United Kingdom	UKCC/BJA level 2	6–10	Regional, national
16	40	United Kingdom	UKCC/BJA level 2	>20	Regional, national
17	49	United Kingdom	UKCC/BJA level 2	11–20	National
18	60	United Kingdom	UKCC/BJA Level 3	>20	Club, regional, national
19	48	Japan	Japanese Level A license	1–5	Club
20	38	Not specified	IJF academy	>20	International
21	48	Not specified	Olympic coach	>20	International
22	43	Not specified	Not specified	Not specified	Regional, national, international
23	41	United Kingdom	UKCC/BJA level 2	Not specified	Not specified

### Coach-reported perceptions and experiences of LOC

The first section asked about recalled observations and perceptions relating to LOC and its consequences (Table 2). Most coaches reported observing LOC less than monthly (19/23) or never (2/23). Perceptions of possible consequences and of similarity to TBI were mixed. The reported technique category and duration responses are perceptions or retrospective estimates, not objective risk or timing data.

**Table 2:** Coach-reported perceptions and experiences concerning LOC associated with shime-waza.

Q. Do you see loss of consciousness related to judo strangles more often in competition or during training?	Competition: 15/23 (65.2%) Training: 2/23 (8.7%) About the same: 4/23 (17.4%) Unsure: 2/23 (8.7%)
Q. How frequently have you seen loss of consciousness to athletes because of a judo strangle?	Every day: 0/23 (0%) A few times a week: 0/23 (0%) Once a week: 0/23 (0%) Less than once a week: 1/23 (4.3%) Once a month: 1/23 (4.3%) Less than once a month: 19/23 (82.6%) Never: 2/23 (8.7%)
Q. Do you think there are any consequences of loss of consciousness from a judo strangle?	Yes: 15/23 (65.2%) No: 7/23 (30.4%) Unsure: 1/23 (4.3%)
Q. In your opinion, which judo strangle technique most commonly leads to loss of consciousness?	Collar-used type: 21/23 (91.3%) Arm-used type: 0/23 (0%) Leg-used type: 2/23 (8.7%)
Q. In your experience, how long does the loss of consciousness last after a judo strangle?	Less than 10 s: 12/22 (54.5%) From 10 to 30 s: 9/22 (40.9%) From 30 s to a minute: 1/22 (4.5%) More than a minute: 0/22 (0%)
Q. Do you believe that loss of consciousness from a judo strangle can be similar to a concussion?	Yes: 8/23 (34.8%) No: 9/23 (39.1%) Unsure: 6/23 (26.1%)

### Coach-reported awareness, perceptions, and practices concerning prevention and management

The second section asked about awareness of protocols, perceptions of available medical support, and observations following LOC (Table 3). Awareness that a protocol exists should not be interpreted as confirmation that a coach knows its content or applies it correctly. These findings identify areas for education and future study rather than the quality or effectiveness of current management.

**Table 3:** Coach-reported awareness, perceptions, and practices concerning prevention and management.

Q. Are you aware of a protocol which is implemented if an athlete loses consciousness from a strangle during competition?	Yes: 18/23 (78.3%) No: 3/23 (13.0%) Unsure: 2/23 (8.7%)
Q. Do you believe there is adequate medical assistance and knowledge of how to deal with loss of consciousness after a strangle in judo competition?	Yes: 15/23 (65.2%) No: 7/23 (30.4%) Unsure: 1/23 (4.3%)
Q. Have you noticed any changes in your athlete's behaviour as a result of being strangled?	Yes: 15/23 (65.2%) No: 7/23 (30.4%) Unsure: 1/23 (4.3%)

## Discussion

In a small sample of judo coaches, this exploratory study identified varied perceptions, recalled observations, awareness, and self-reported practices with regards to LOC associated with shime-waza. These findings should not be interpreted as a measure of knowledge, an estimate of the frequency of LOC in judo, or evidence of the safety or effectiveness of specific management practices.

### Coach-reported perceptions and experiences

Most coaches reported recalling LOC more often in competition than in training. Although submission should prompt release of a technique before LOC occurs, the competitive setting may influence the willingness of players to submit, as doing so would end the contest. However, these responses do not assess the relative occurrence of LOC in competition or training. The questionnaire did not collect exposure data such as observation time, coaching hours, numbers or levels of players coached, competition exposure, frequency of shime-waza practice, or a defined recall period. This should therefore be interpreted only as reflecting how frequently coaches recalled observing LOC. Moving forward, video-based competition research may provide a more appropriate source for observed timing and frequency than retrospective estimates (4).

Perceptions of possible consequences were mixed, which is more appropriately interpreted as evidence of uncertainty and potential educational needs as opposed to evidence that LOC is either harmless or harmful. Although prolonged cerebral hypoxia can result in hypoxic-ischaemic brain injury, such an outcome would be expected to require sustained or inadequately terminated compression. In regulated judo, this has been argued to be plausible only in circumstances in which ‘the competitors or referee were acting in a completely nonstandard fashion’ (12, 15).

Coaches also displayed a varied understanding of the similarities between LOC from shime-waza and TBI. Acute LOC after shime-waza and TBI are not equivalent clinical entities: the former is generally associated with transient neck compression, whereas TBI is caused by biomechanical forces and can occur with or without LOC (5). This distinction matters because the mechanisms, clinical context, and management pathways differ. The 26.1% of participants unsure whether LOC following shime-waza could be similar to TBI clearly illustrates uncertainty among coaches. Unlike TBI, evidence is currently insufficient to determine the long-term neurological consequences of repeated shime-waza exposure (16). Thus, an absence of demonstrated long-term harm should not imply evidence of safety. Acute symptoms reported after recovery in young players have included transient confusion or disorientation, limb symptoms, and dizziness, with some symptoms not resolving immediately (17). Ultimately, any athlete who experiences LOC requires appropriate assessment, observation, and a cautious return to play.

### Awareness, perceptions, and self-reported practices

Most coaches were aware that a protocol existed for LOC. British Judo Association guidance states that players must be removed from play immediately and are advised to rest briefly, although a graduated return-to-play is not always required (18). Awareness that a protocol exists, however, does not necessarily establish knowledge of its content, implementation, or effectiveness. Regulations and post-LOC requirements may also differ by governing body, age category, competition, and time period, meaning that coaches and organisers must refer to the applicable current rules and corresponding medical guidance. Responses about the adequacy of medical assistance represent coaches' perceptions rather than an audit of medical provision. Nevertheless, variation in these responses highlights the importance of clearly defined escalation pathways, access to appropriate medical assessment, incident reporting, and education for coaches and other personnel involved in athlete safety.

Immediate release of a given technique when submission or LOC is recognised is fundamental. Any player who does not rapidly regain normal responsiveness, has persistent confusion, deteriorating conscious state, seizure activity, new focal neurological symptoms, persistent visual disturbance, or suspected neck injury requires urgent medical assessment (19). A person with a suspected spinal cord injury should not be moved unnecessarily unless this is required to manage an immediate threat to life (20). Reports of behavioural change following LOC in the present survey were retrospective observations made by coaches and cannot establish the presence, absence, severity, or duration of clinical symptoms.

### Safety considerations

For historical reference, Katsu or Kuatsu refers to traditional resuscitative practices by the Kodokan associated with recovery after a choke. These practices are not considered as an alternative to contemporary evidence-based medical assessment or emergency management (3).

Although serious events appear uncommon in supervised judo, the available evidence does not yet confer a zero-risk environment. The proper application of judo chokes has historically been regarded as unlikely to result in significant injury, and serious neurological complications reported within the sport are rare: only one case of carotid artery occlusion and right middle cerebral artery territory infarction has been recorded in the sport (21). Reports of fatal neck restraint outside organised sport are not directly transferable to regulated training or competition, where supervision, rules, and prompt release of techniques meaningfully alter the context (11). Nevertheless, such reports illustrate the potential consequences of sustained or improperly applied neck compression and reinforce the importance of conservative safety measures.

It would therefore be inappropriate to suggest that comparable events are likely to occur during properly supervised judo. Instead, the relevant implication is that safe practice depends on multiple safeguards operating together. These include technical instruction, precise execution and control of shime-waza, qualified supervision, and ongoing education for both coaches and players (11, 14). Restrictions on the use of shime-waza in younger age categories and in Veteran players (≥60 years) provide an additional layer of risk reduction.

The findings of this study identify potential areas in which further education concerning LOC associated with shime-waza is likely to be beneficial. Future work should extend beyond coaches to examine the knowledge, experiences, and attitudes of players themselves. Prospective, exposure-based studies would also be better placed to establish how often LOC occurs, the circumstances in which it arises, and whether repeated exposure is associated with clinically significant outcomes.

## Limitations

This study has several important limitations. The study-specific questionnaire was not formally validated, and no score or criterion was used to classify knowledge. The sample used was small and predominantly United Kingdom-based, and all participants were male: these factors limit diversity and subgroup comparison. The English-only questionnaire may also have restricted participation from Greece and Japan. Location and qualification data were incomplete for some participants, and equivalence across national systems could not always be fully verified. The sample was also heterogeneous with respect to self-reported coaching qualifications, years of coaching experience, and coaching level. This may have contributed to variability in responses and further limits subgroup interpretation. Findings were self-reported and therefore susceptible to selection, recall, and interpretation bias. The questionnaire did not provide an operational definition of LOC, and several items asked about perceptions rather than objectively verifiable knowledge. Additionally, free-text answers were not subjected to formal qualitative analysis. These constraints preclude causal inference or assessment of the quality or effectiveness of safety practices.

## Conclusion

This exploratory study describes varied coach-reported perceptions, experiences, awareness, and practices surrounding LOC and shime-waza. It does not measure knowledge levels, estimate the occurrence of LOC, or establish the effectiveness of current safety practices. The findings identify potential areas in which further education for coaches and players may be useful and highlight the importance of appropriate safety processes, recognition and medical assessment of LOC, and future prospective research using validated instruments.

## Data Availability

The original contributions presented in the study are included in the article/Supplementary Material, further inquiries can be directed to the corresponding author.
