# Supplementary material for: Loss of consciousness associated with Shime-Waza in judo: an exploratory study of coaches' perceptions, experiences, and practices
Source: Front Sports Act Living. 2026 Oct 23;8:1955805. doi: 10.3389/fspor.2026.1955805 (PMC13739811; doi:10.3389/fspor.2026.1955805)
Supplement: Supplementary file 1 [file Supplementaryfile1.docx]

# Judo coaches’ opinions on judo strangles (*shime-waza*) as a possible injury risk:

# A Questionnaire-Based Study.

Name:

# Participant knowledge of injury risk

1. **Do you see loss of consciousness related to judo strangles more often in competition or during training?**

Competition

Training

About the same

Unsure

2. **How frequently have you seen loss of consciousness to athletes because of a judo strangle?**

Every day

A few times a week

Once a week

Less than once a week

Once a month

Less than once a month

Never

3. **Do you think there are any consequences of loss of consciousness from judo strangle?**
4. **If answered yes, then which?**
5. **What types of consequences you have witnessed following a judo strangle leading to unconsciousness?**
6. **In your opinion, which judo strangle technique most commonly leads to loss of consciousness?**

Collar-used type (Katate-jime, Okuri-eri-jime, etc)

Arm-used type (Hadaka-jime, Juji-jime, etc)

Leg-used type (Sankaku-jime)

7. **In your experience, how long does the loss of consciousness last after a judo strangle?**

Less than 10 second

From 10 to 30 second

From 30 second to a minute

More than a minute

8. **Do you believe that loss of consciousness from a judo strangle can be similar to a concussion?**

Yes

No

Unsure

9. **If answered yes, then why?**
10. **Do you think there are any consequences of loss of consciousness for brain damage?**
11. **Do you think there are any consequences of loss of consciousness for young athlete especially in mental or development?**

# Participant knowledge of prevention and management

12. **Are you aware of a protocol which is implemented if an athlete loses consciousness from a strangle during competition?**

Yes

No

Unsure

13. **If yes, can you briefly describe a protocol which is implemented if an athlete loses consciousness from a strangle during competition?**
14. **Do you follow a protocol for your athletes regarding return to practice after loss of their consciousness from judo strangle?**
15. **Do you believe there is adequate medical assistance and knowledge of how to deal with loss of consciousness after a strangle in judo competitions?**

Yes

No

Unsure

16. **Can loss of consciousness be prevented during competitions? If yes, then how?**
17. **Have you noticed any changes in your athlete’s behaviour as a result of being strangled?**

Yes

No

Unsure

18. **If yes, please state the changes.**
19. **Please state any proposals you have in order to prevent loss of consciousness during a judo strangle.**

# Participant information

20. **What is your age?**
21. **What is your gender?**

Female

Male

Non-binary

Transgender

Intersex

Type own response

Prefer not to say

22. **Which region of the UK are you currently based in?**

England

Scotland

Wales

Northern Ireland

Other

23. **What level of judo coaching qualification do you have?**

UKCC / BJA Level 1

UKCC / BJA Level 2

UKCC / BJA Level 3

UKCC / BJA Level 4

EJU Level 3

EJU Level 4

EJU Level 5

EJU Level 6

IJF Academy

Other (please specify)

24. **How long have you been a qualified judo coach?**

<1 year

1-5 years

6-10 years

11-20 years

20+ years

25. **What level of competition do your athletes participate in?**

Club

Regional

National

International

Other (please specify)

26. **What is your Dan grade?**
